# Relevance of a Mobile Internet Platform for Capturing Inter- and Intrasubject Variabilities in Circadian Coordination During Daily Routine: Pilot Study

**DOI:** 10.2196/jmir.9779

**Published:** 2018-06-11

**Authors:** Sandra Komarzynski, Qi Huang, Pasquale F Innominato, Monique Maurice, Alexandre Arbaud, Jacques Beau, Mohamed Bouchahda, Ayhan Ulusakarya, Nicolas Beaumatin, Gabrièle Breda, Bärbel Finkenstädt, Francis Lévi

**Affiliations:** ^1^ Cancer Chronotherapy Team School of Medicine University of Warwick Coventry United Kingdom; ^2^ European Associated Laboratory of the Unité Mixte de Recherche Scientifique 935 Institut National de la Santé et de la Recherche Médicale Villejuif France; ^3^ Cancer Chronotherapy Team Department of Statistics University of Warwick Coventry United Kingdom; ^4^ Department of Oncology North Wales Cancer Treatment Centre Bodelwyddan United Kingdom; ^5^ Department of Oncology Paul Brousse Hospital Assistance Publique-Hôpitaux de Paris Villejuif France; ^6^ Altran Research Vélizy-Villacoublay France

**Keywords:** circadian clock, eHealth, temperature rhythm, rest-activity rhythm, time series analyses, domomedicine, biomarkers

## Abstract

**Background:**

Experimental and epidemiologic studies have shown that circadian clocks’ disruption can play an important role in the development of cancer and metabolic diseases. The cellular clocks outside the brain are effectively coordinated by the body temperature rhythm. We hypothesized that concurrent measurements of body temperature and rest-activity rhythms would assess circadian clocks coordination in individual patients, thus enabling the integration of biological rhythms into precision medicine.

**Objective:**

The objective was to evaluate the circadian clocks’ coordination in healthy subjects and patients through simultaneous measurements of rest-activity and body temperature rhythms.

**Methods:**

Noninvasive real-time measurements of rest-activity and chest temperature rhythms were recorded during the subject’s daily life, using a dedicated new mobile electronic health platform (PiCADo). It involved a chest sensor that jointly measured accelerations, 3D orientation, and skin surface temperature every 1-5 min and relayed them out to a mobile gateway via Bluetooth Low Energy. The gateway tele-transmitted all stored data to a server via General Packet Radio Service every 24 hours. The technical capabilities of PiCADo were validated in 55 healthy subjects and 12 cancer patients, whose rhythms were e-monitored during their daily routine for 3-30 days. Spectral analyses enabled to compute rhythm parameters values, with their 90% confidence limits, and their dynamics in each subject.

**Results:**

All the individuals displayed a dominant circadian rhythm in activity with maxima occurring from 12:09 to 20:25. This was not the case for the dominant temperature period, which clustered around 24 hours for 51 out of 67 subjects (76%), and around 12 hours for 13 others (19%). Statistically significant sex- and age-related differences in circadian coordination were identified in the noncancerous subjects, based upon the range of variations in temperature rhythm amplitudes, maxima (acrophases), and phase relations with rest-activity. The circadian acrophase of chest temperature was located at night for the majority of people, but it occurred at daytime for 26% (14/55) of the noncancerous people and 33% (4/12) of the cancer patients, thus supporting important intersubject differences in circadian coordination. Sex, age, and cancer significantly impacted the circadian coordination of both rhythms, based on their phase relationships.

**Conclusions:**

Complementing rest-activity with chest temperature circadian e-monitoring revealed striking intersubject differences regarding human circadian clocks’ coordination and timing during daily routine. To further delineate the clinical importance of such finding, the PiCADo platform is currently applied for both the assessment of health effects resulting from atypical work schedules and the identification of the key determinants of circadian disruption in cancer patients.

## Introduction

### Background

Circadian (about-24-hour) rhythms regulate mammalian physiology, as well as cell metabolism, proliferation, and survival over the 24 hours. These rhythms play an important role in disease processes and treatment effects, which has been largely overlooked in medicine [1-3]. They are generated at single-cell level by molecular clocks, consisting of interwoven feedback loops involving transcription-translation of 15 known specific *clock* genes including Bmal1, Clock, Per2, and Rev-erbα [4]. The molecular clocks are coordinated at the whole organism level by the suprachiasmatic nuclei, a hypothalamic pacemaker, which also helps circadian rhythms adjust to light-dark and other environmental 24-hour cycles through the rhythmic control of rest-activity, body temperature, feeding, as well as cortisol and melatonin secretions [1-4]. Thus, both glucocorticoids and body temperature rhythms reset molecular clocks and cellular circadian rhythms in vitro and in vivo [5-8].

Rhythm studies in humans have assumed similar circadian synchronization among subjects, thus inferring the reliability of transverse sampling of different subjects at different time points over 24 hours, and using average values for describing circadian patterns in the group or the population [9]. Treatment effects could also differ according to circadian timing or chronomodulated scheduling of medications in a consistent fashion across individual subjects with a similar circadian entrainment [1,10-12]. Such standardized approaches to chronotherapy proved valid in experimental rodents of same sex, strain, and age, which were synchronized with the same alternation of 12-hours of light and darkness, especially for anticancer drugs [1,13]. This was also true for healthy subjects maintained in human physiology laboratories under controlled environmental conditions [14]. However, little is known regarding circadian rhythms in individual healthy humans or patients during their daily routine.

Intersubject variability in circadian phase has been suggested, based on chronotype questionnaires administered to large populations of presumably healthy subjects [15]. Intersubject differences in both daily timing and 24-hour pattern have also been shown in individual patients collecting up to 5 daily samples of salivary cortisol and/or melatonin determinations for up to 2 days [16-18]. The limitations resulting from such low sampling frequency were overcome through rest-activity monitoring, using a wrist watch accelerometer for a few days to a few weeks [19-21]. The rest-activity time series led to identify the dichotomy index I<O, the relative amount of activity in bed that was below the median activity out of bed, as an independent predictor of progression-free survival and overall survival among 436 patients with metastatic colorectal cancer [22]. Most importantly, the patients whose I<O was below the median value of 97.5% had a median survival of 11.9 months, as compared with 21.6 months for those with an I<O index over 97.5% [22]. The I<O was also negatively associated with fatigue and appetite loss, and positively with health-related quality of life as assessed by both the European Organization for Research and Treatment of Cancer Quality of Life Questionnaire C30 EORTC QLQ-C30 and the MD Anderson Symptom Inventory questionnaires in cancer patients with locally advanced or metastatic disease [23,24]. Furthermore, circadian rest-activity disruption, as measured with an I<O of 97.5% or less, in patients receiving cancer chemotherapy could indeed represent an early warning signal of deterioration and emergency hospitalization [25,26]. However, I<O values did not correlate with sex, both in healthy subjects or in cancer patients, or with efficacy of a standardized chronomodulated chemotherapy protocol in cancer patients [19,20,27], whereas the latter profoundly differed between men and women [28]. These clinical data stressed the need for the combination of rest-activity with circadian temperature biomarker to gather more reliable estimates of the circadian phase and to personalize the timing of chronotherapy.

Indeed, despite their consistent and reproducible clinical relevance, the rest-activity time series provide imprecise estimates regarding circadian phase, due to both its square-wave 24-hour pattern and the strong masking effect of the societal routine on the endogenous activity rhythm. Predominant low values of activity suggest prolonged periods of rest during nighttime, whereas frequent high intensity activity occurs during the day, with substantial within-day and day-to-day variability [29]. Nevertheless, a first fixed electronic health (eHealth) internet platform was developed within the inCASA European project—combining telemetric activity monitoring with self-rated symptoms and self-measured body weight. Testing of the platform in 31 cancer patients on chronotherapy at home demonstrated a per protocol compliance rate of ~85% over 1 month, and enabled prediction of emergency hospitalization due to treatment toxicity 3 days in advance [26].

Precise information regarding circadian phase and circadian coordination is also critical for the appropriate timing of treatment delivery to reduce adverse events and/or enhance efficacy [1,10-13,30]. Moreover, both diseases such as cancer and treatments can disrupt the circadian timing system (CTS) and result in associated symptoms and reduced survival, especially in cancer patients [31]. To address these issues, we have designed an upper chest e-sensor that records and teletransmits activity, temperature and tri-axial orientation. This sensor is integrated into a novel eHealth platform (PiCADo). The circadian rhythms in core and skin surface temperature of men are usually 8-12 hours out of phase, with respective maxima occurring near 16:00 at day time, and near 2:00 at night [32]. The early night drop in core body temperature results from the vasodilatation of the skin vessels and associated rise in skin surface temperature [33]. Such temperature changes are critical for triggering the onset of sleep [34]. The site of temperature measurements for achieving continuous and noninvasive, yet reliable, assessment of human body temperature rhythms in real life has been a challenge over the past decades. The use of a rectal probe has been discouraged as a result of the risk of rectal perforation [35]. Axillary and wrist skin surface temperature records were shown to be largely contaminated by changes in environmental temperature [36-39]. The recent availability of an oral temperature pill has enabled the continuous recording of internal body temperature, yet only for durations that match the gastro-intestinal transit time, ie, ~24-48 hours [40,41]. Previous work by others highlighted the reduced influence of environmental temperature changes on skin surface temperature measurements taken at the upper-anterior chest wall [42,43]. We confirmed these findings through combining infrared technology with continuous recording of patched temperature sensors [44,45]. We further developed dedicated statistical methods to compute dynamic changes in rhythm parameters by combining the inference methods for obtaining interval estimates based on spectral bootstrap with time-varying spectral estimation [46,47].

### Objectives

Here, we assessed—for the first time—the performance and relevance of the PiCADo platform for capturing inter-and intrasubject variabilities in the CTS both during daily routine and in real time. We hypothesized that the combination of rest-activity and temperature monitoring would identify large interindividual differences in circadian coordination. The latter would notably support the personalized adaptation of the optimal timing of medications to jointly minimize treatment morbidities and enhance efficacy.

## Methods

### Study Design

**Figure 1:**
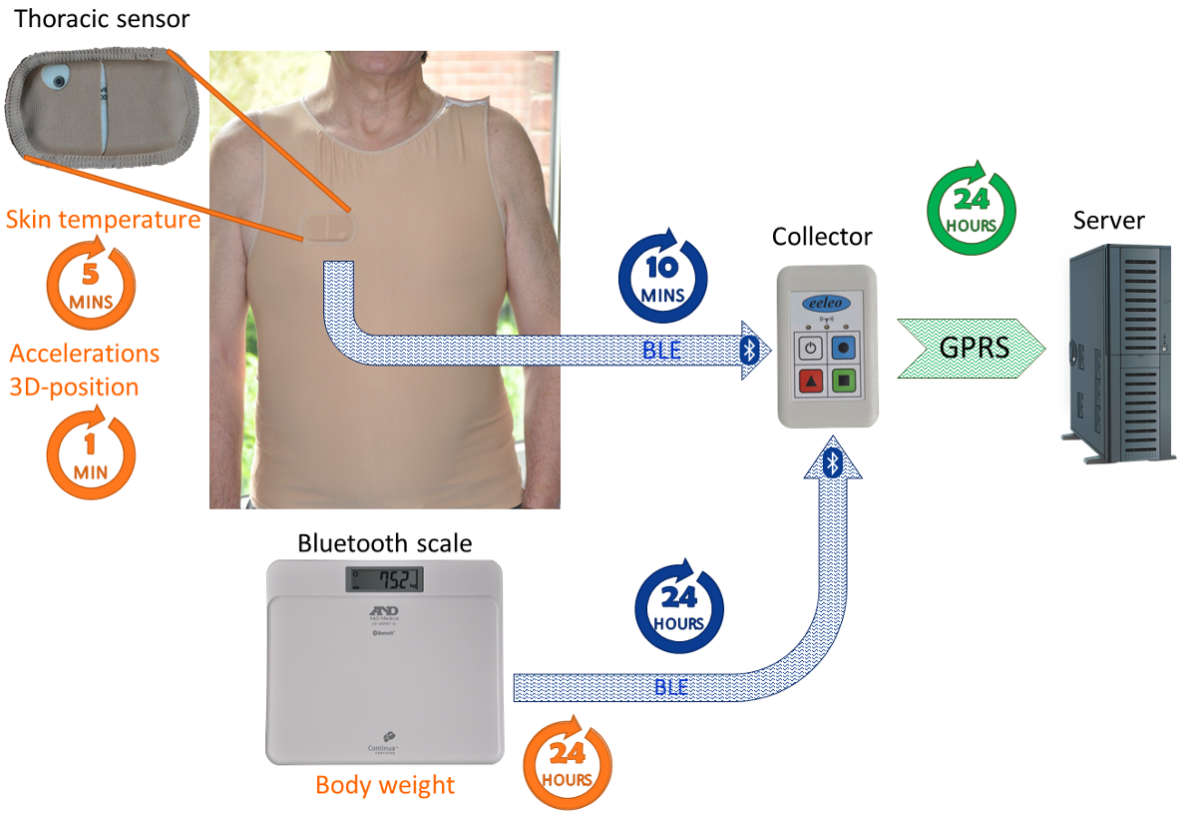
eHealth domomedicine platform technology. The chest sensor embedded into the vest shirt with an open area for infrared temperature measurements is shown in the upper left corner. The epoch length of the data points as well as length of time intervals between teletransmission events are indicated within each circle for each variable. Teletransmissions involve Bluetooth Low Energy (BLE) from sensor to gateway and General Packet Radio Service (GPRS) from gateway to server, from which data can be retrieved continuously.

The main objective was to determine whether any inter- and intrasubject differences in human circadian coordination could be captured in real time through remote and noninvasive real-time monitoring during the subjects’ usual routine. Such a goal represented a critical step toward the personalization of treatment timing according to individual circadian rhythms, especially for cancer therapies. A new mobile eHealth platform (PiCADo) was designed on purpose. The PiCADo specifications were defined within several multidisciplinary and multiuser focus groups involving nurses, medical oncologists, general practitioners, biomedical and informatics engineers, socio-anthropologists, and chronobiologists, and through analyzing elderly people’s responses in living labs. Three parameters—activity, temperature, and position— are measured using a single CE-marked chest sensor emitter (Movisens, Karlsruhe, Germany) and a pocket-sized CE-marked gateway (Eeleo, Montrouge, France), which could gather further information from other Bluetooth (BT)- and Bluetooth Low Energy (BLE)-connected devices, and send them to a server via the general packet radio service (GPRS) at the required frequency, which may be tuned down as low as every hour, in case of measurements of preset emergency values (Figure 1). This latter function was not activated here. Thus, the PiCADo platform consisted of a chest sensor that measured skin surface temperature every 5 min, the number of accelerations, and the orientation in 3 dimensions every min. All data were teletransmitted via BLE to a pocket-sized gateway, which also could gather data measured by other connected Bluetooth and BLE devices, such as a weight scale. The gateway sent all data to a server every 24 hours. Three cohorts of people were involved, each with different specifications regarding observation span (4 days vs 7-30 days), sensor-carrying method (patch vs dedicated vest or bra), and health condition (healthy vs cancer). Subjects in cohorts 1 and 2 had to be 21 years or older, display no active disease, and not work at night. The study was planned without any intervention. Subjects were advised to remove the sensor for around 20 min once per day to avoid contact with water during showering. The study was conducted according to the Helsinki Declaration [48]. All the subjects enthusiastically volunteered and provided informed consent for carrying and testing the platform system.

### Data Management

All anonymous electronic data were transferred from the gateway at home to the server according to the GPRS communication protocol. Once on the server, the data were stored based on HL7 standards (international standards for transfer of clinical and administrative data). Data were downloaded from the server to the computer of our biomedical engineer, who was the only person having access to the server. Data were saved on a secure storage server according to the national Data Protection and Freedom of Information Acts guidance. Data transmission was inspected at least twice per week during the monitoring sessions to insure the proper functioning. Data were retrieved and processed at the end of the monitoring.

### Statistical Methods

#### Treatment of Missing Data and Data Preprocessing

Times at which subjects removed the sensor were identified retrospectively by noting a string of zero Position and Activity counts, jointly with corresponding temperature measurements decreasing toward room temperature values. These intervals were marked as missing values. To perform spectral analysis, the raw activity and temperature data were aggregated over hourly intervals and corresponding median values were computed. The hourly interval length provided a good resolution of the periodogram at frequencies of interest, especially those corresponding to the circadian range. The endpoints of the hourly aggregated data were connected using linear interpolation in case of recording gaps ≤7 hours. For gaps >7 hours, as it did occur for 7 subjects, the recordings of the whole corresponding 24 hours were ignored for purposes of the analyses.

#### Estimation of Circadian Parameters

The Spectrum-Resampling (SR) algorithm [46] was applied to estimate the circadian parameters of interest, namely, period, amplitude, and phase of the first and second largest peaks in the spectra for both rest-activity and temperature time series. This method first identified the most important frequencies and their corresponding periods from the estimated spectrum, then fitted a Fourier-type regression model to the data, to obtain the corresponding amplitudes and phases. The SR method provided a bootstrap framework, where all circadian parameters were estimated as the median of the bootstrap samples, and their 90% central confidence intervals were approximated by the corresponding percentiles of the bootstrap samples. To analyze the intrasubject variability over time, the same methodology of spectral analysis was performed over moving windows of 3 days each, with a 1-hour shift per spectral estimate [49]. We note that the window length should be at least 2-3 days, as at least 2 full cycles are needed to estimate the period length.

#### Regression Analysis

Multivariate regression analysis was applied to test for the effects of covariates such as sex, age, weight, and cohort. As response variable Y, we considered a selection of estimated circadian parameters that summarized the behavior of the biomarkers for each subject, including (1) the amplitude of the main period of temperature, which was obtained from spectral estimation; (2) the amplitude of activity, during prolonged (usually daily) activity spans, as approximated by the interquartile range (IQR) or amplitude of 50% central values of observed values; (3) the spectral gravity center (or mean period) of temperature; and (4) the spectral gravity center (or mean period) of rest-activity. Possible explanatory variables were sex, age, weight, and amplitude of daily activity as given by the IQR of daily activity counts, cohort, and the following interaction terms: sex*age, sex*weight, and sex*IQR of daily activity. Model selection was performed stepwise (as implemented in R function and based on Akaike’s information criterion) [50]. Significance of explanatory variables was tested by *t* tests of the corresponding coefficients—where a significant effect was concluded for *P* values smaller than .05. As 0 and 1, respectively, encoded for females and males, a sex-specific effect was computed in a straightforward way in that a sex-specific influence of a covariate was concluded, if the coefficient corresponding to the interaction term sex*covariate was significant in the regression.

## Results

### Subject Characteristics and Study Conduct

**Figure 2:**
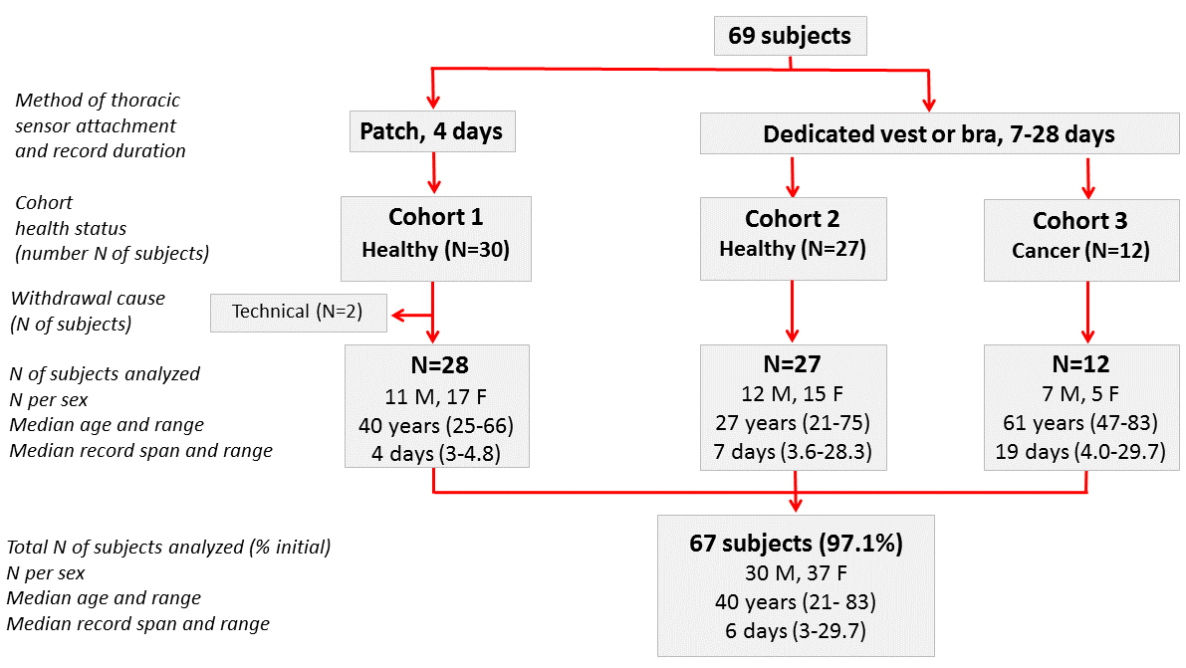
Consort diagram. The 69 subjects were enrolled in 1 of 3 cohorts that differed according to the method of sensor attachment and record duration (first row), health status (second row), and number of subject per group, including sex, age (median and range), and record duration (median and range).

The PiCADo Domomedicine platform was tested by 69 people. Assessable time series of the 3 variables were obtained for 67 of 69 subjects (97%) over a median duration of 6 days (1st-3rd quartiles, IQ, 4.0 to 12.1), ranging from 3 to 29.7 days. A total of 30 males and 37 females, aged 21 to 83 years, participated in 1 of 3 cohorts of subjects (Figure 2; Table 1).

All subjects were asked to keep their usual daily routines, besides carrying the sensor day and night for the whole monitoring duration, and keeping the gateway within a distance of 2 m. The sensor was initially patched onto the upper left anterior thorax of 28 healthy subjects in Cohort 1, using Tegaderm (10x12 cm, 1626W, 3M^T^, Diegem, Belgium) for a median duration of 4 days. To avoid the use of the potentially irritating patches for longer durations, a dedicated vest and bra were designed, which could properly lodge the sensor (Thuasne Medical, Saint Etienne, France). This system was tested by 27 healthy subjects in 2 countries for durations ranging from 3.6 to 28.3 days (Cohort 2). The platform was used by 18 subjects in the United Kingdom for a median duration of 7 days (Cohort 2.1), and by 9 subjects in France, jointly with a BLE weight scale, for a median duration of 19 days (Cohort 2.2; Table 1). To probe the platform for a prolonged use in patients with cancer, the sensor, cloth, and gateway system was further assessed in 12 patients with advanced or metastatic cancer for a median duration of 18.5 days (Cohort 3, see characteristics in Multimedia Appendix 1).

### Inter- and Intrasubject Differences Captured by Time Series Analyses

**Figure 3:**
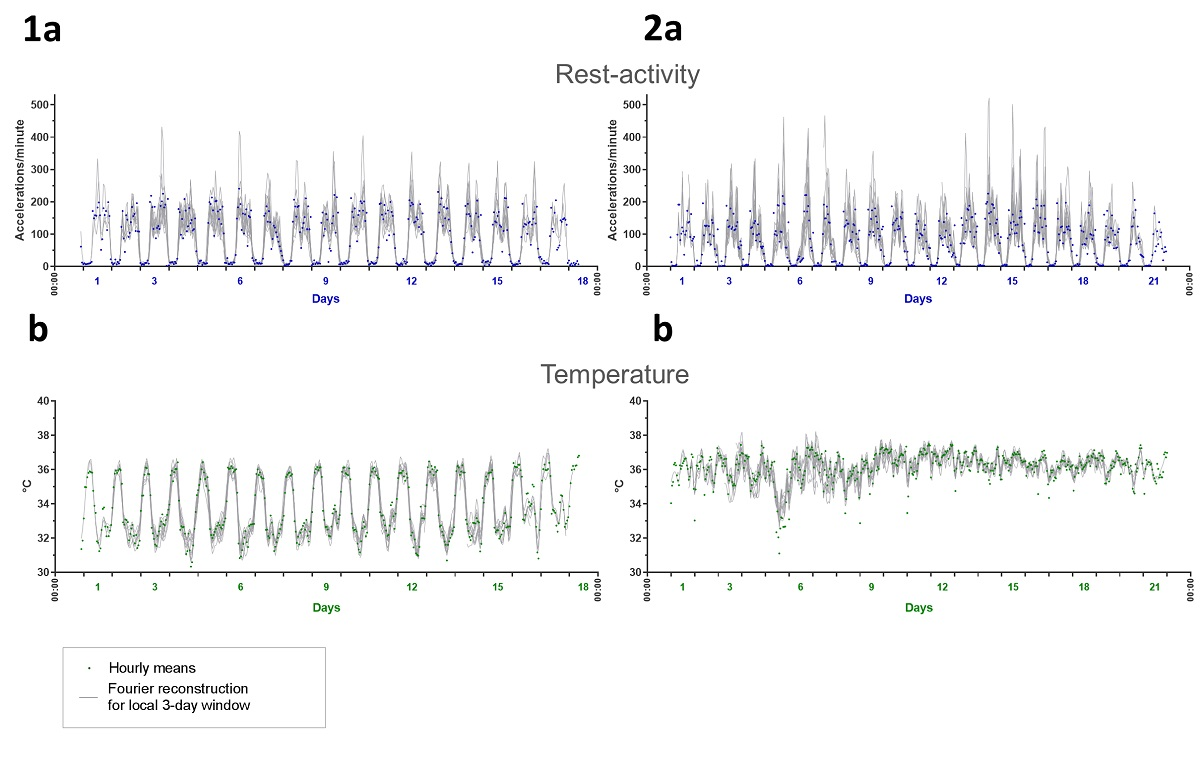
Inter- and intrasubject variability in circadian patterns illustrated by chronograms of rest-activity (a) and thoracic skin surface temperature (b) of 2 healthy subjects. Panel 1 (left): time series from a 57-year-old female researcher, with usual respective times of awakening and retiring at 8:30 and 22:30; mean rest-activity I<O of 99.7%, with intrasubject coefficient of variation of 0.2%. Panel 2 (right): time series from a 27-year-old female student, usually awakening at 8:40 and retiring at 01:00; mean I<O of 99.2%, with a coefficient of variation of 1.2%.

**Figure 4:**
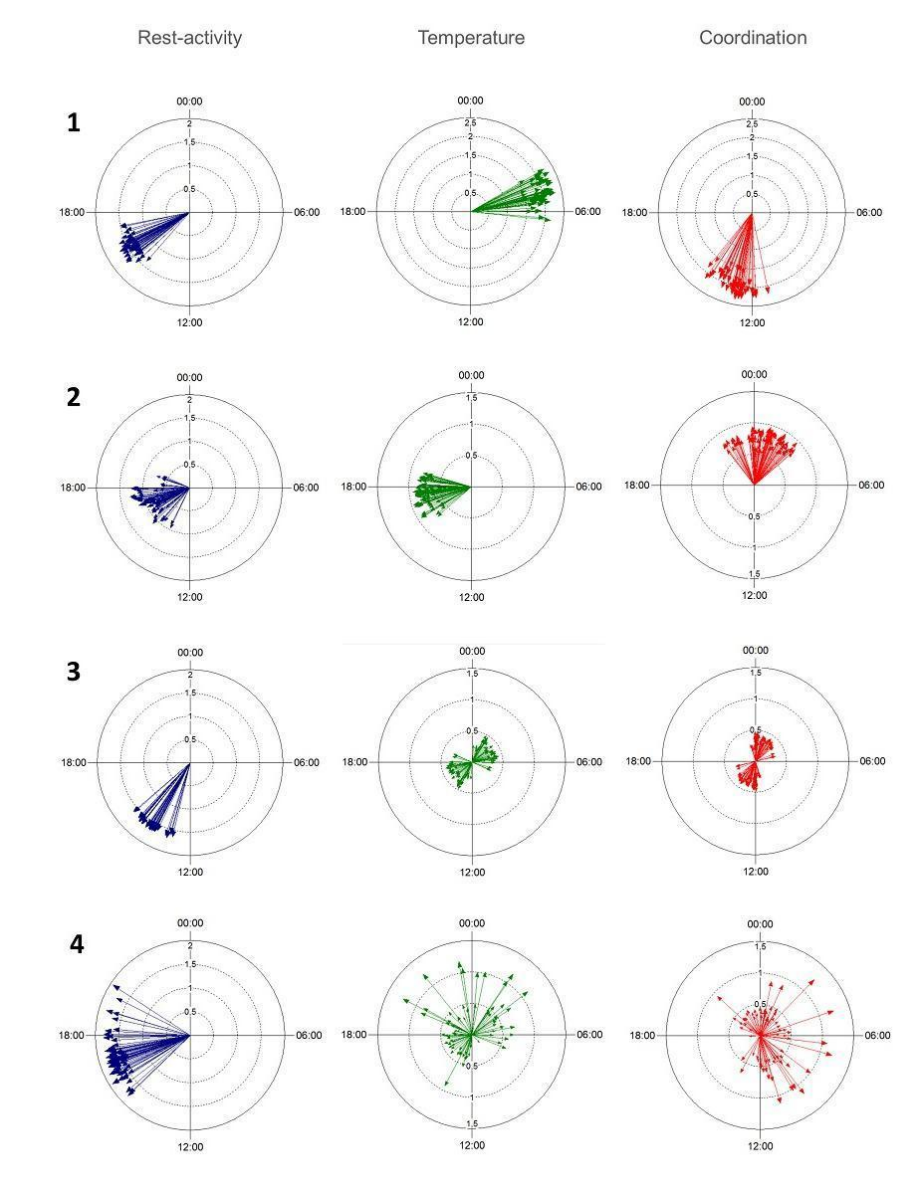
Inter- and intrasubject variability in circadian acrophases and amplitudes of rest-activity (left panels), temperature (middle panels), and circadian coordination (right panels). Illustrative examples through polar plots in 4 healthy subjects, whose 3-day time series shifted by 6 hours (subjects 1 and 4) or 1 hour (subjects 2 and 3), have been analyzed using the sampling-resampling spectrum analysis. The length of each vector represents the amplitude of the dominant period and its direction points toward the timing of the corresponding acrophase.

Time series were preprocessed to account for missing data (see Material and Methods). Using the spectrum resampling method, period, amplitude, and acrophase corresponding to the largest (fundamental) and, if significant, second-largest peak in the spectrum were estimated, along with their respective 90% Confidence Limits, for each subject over the whole time span [46]. Time-varying features of the spectrum were also estimated, through the application of the same method to 3-day windows, which were moved along each time series with 1-hour shifts. The clinically relevant Dichotomy Index I<O was further computed, over consecutive 3-day spans, with 24-hour shifts [26,51]. Strikingly different circadian patterns, rhythm parameters, and I<O values were identified among the 55 healthy subjects (Figures 3 and 4). Although the rest-activity pattern remained consistent from 1 day to the next in the 55 healthy subjects, the temperature pattern varied from day to day in 16 of them, as revealed by changes over time in the dominant periods and the amplitude-acrophase vectors (Figure 4, subjects 3 and 4). Thus, our methodology revealed and quantified day-to-day changes in circadian parameters, thereby enabling the determination of circadian variability both within and between subjects during real-life conditions.

**Table 1:** Subjects and records characteristics. Median and distribution of data for all quantitative variables. M/F refers to male/female.

Cohort	Total N (M/F)	Age, years (range) [25-75% IQ^a^]	Weight, kg (range) [25-75% IQ]	Height, cm (range) [25-75% IQ]	BMI, kg/m^2^ (range) [25-75% IQ]	# of subjects with partial record (cause)	Valid time series duration, days (range) [25-75% IQ]	Missing data, days (%) (range) [25-75% IQ]
1	28 (11/17)	40 (25-66) [32.7-49.5]	73 (44-93) [61-77.5]	—	—	0	4.0 (3.0-4.8) [3.9-4.0]	0 (0.8) (0-0.3) [0-0.1]
2.1	18 (8/10)	26 (21-75) [24-32.3]	73 (46-93) [66.7-78]	172 (156-183) [168.5-175.5)]	24 (19-28) [22.2-26.1]	1 (charger dysfunction)	7.0 (3.6-12.4) [7.0-7.4]	0.1 (1.5) (0-0.6) [0.05-0.15]
2.2	9 (4/5)	34 (25-57) [27-38]	71 (54-83) [65-72]	169 (155-195) [162-171]	24 (18-31) [23.3-26]	3 (subject-related)^b^ 1 (unsticking of electronic circuit)	19.0 (4.9-28.3) [17.0-21.7]	2.3 (7.7) (0.3-7.7) [1.1-2.6]
2	27 (12/15)	27 (21-75) [24.5-37.5]	70 (46-93) [65-77]	171 (155-195) [165-174]	24.3 (17.9-31.2) [22.3-26.2]	3 subject-related 2 technical failures	7.4 (3.6-28.3) [7.0-15.3]	0.1 (2.0) (0-7.7) [0.1-1.0]
3	12 (7/5)	61 (47-83) [54-66.5]	66 (45-80) [60-72]	170 (152-185) [164-176]	22.8 (18.5-26.1) [21.4-23.9]	5 (subject-related)^c^	20.3 (4.7-29.7) [16.5-27.2]	1.2 (5.0) (0-20.4) [0.7-2.7]

^a^IQ: interquartile.

^b^Forgetfulness after charging (N=1), travel abroad starting before end of recording span (N=1), wrong charging procedure applied (N=1).

^c^Wrong charging procedure applied (N=2); poor tolerability of adjusted sensor-dedicated cloth due to no current use of bra (N=1), or treatment-related itching (N=1); need for more feedback and support (N=1).

**Figure 5:**
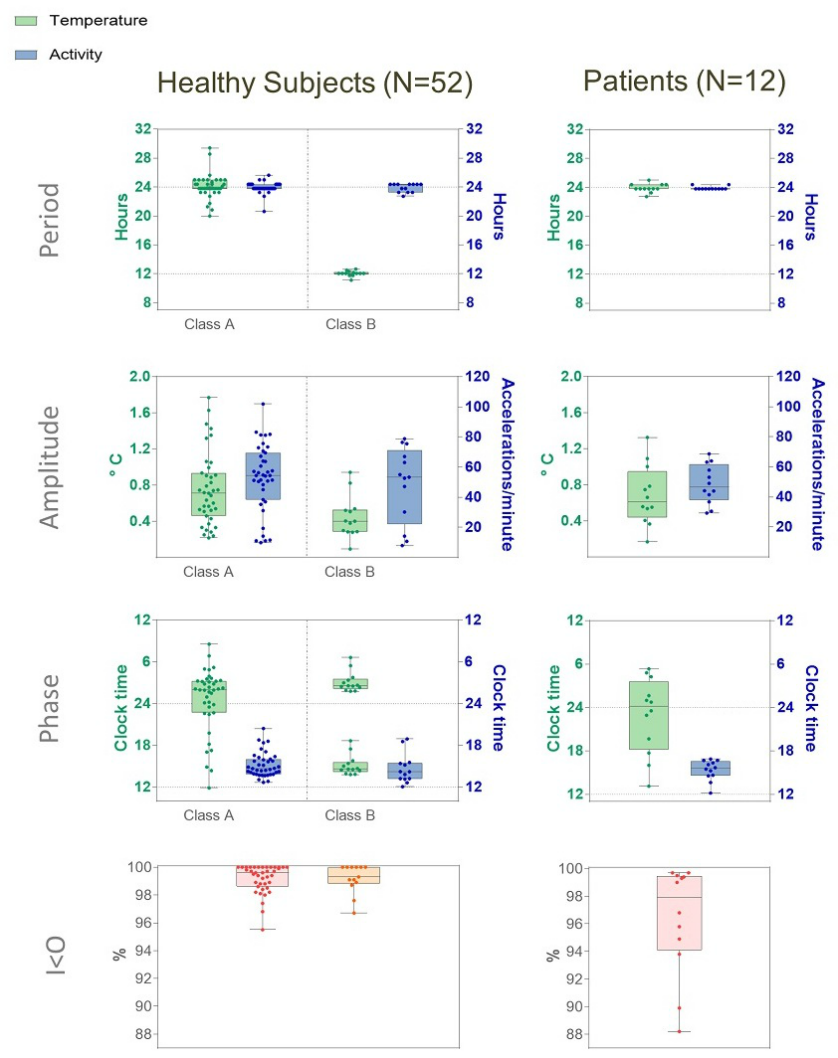
Intersubject variabilities in main rhythm parameters of healthy subjects (left columns) and cancer patients (right column). Median, interquartiles, range, and individual values of dominant periods and corresponding amplitudes and acrophases of temperature (green) and rest-activity (blue), based on spectral analysis of time series over the whole time span.The bottom row depicts the distribution of the dichotomy index I<O of the rest-activity pattern in Class A or B healthy subjects, as well as in cancer patients.

### Subjects Classification According to Temperature Periods and Circadian Coordination

Results of the spectral analyses over the complete time series of the 28 subjects in Cohort 1 were very similar to those of the 27 subjects in Cohort 2 (see Multimedia Appendices 2 and 3). For instance, a dominant circadian rhythm in rest-activity was identified for all subjects, yet with a dominant circadian rhythm in skin surface temperature for 68% (19/28) of the subjects in Cohort 1 and 74% (20/27) of those in Cohort 2; the dominant temperature period was about 12 hour (circa-hemidian) for 25% (7/28) and 22% (6/27) of the subjects in Cohorts 1 and 2, respectively. Atypical patterns were found for 2 and 1 subjects, respectively.

Both cohorts of healthy subjects were pooled, so as to further examine the relations between summary statistics of the temperature and activity variables and their spectral properties, as well as the relevance of available covariates, such as sex, age, and weight in the 55 healthy subjects. The differences between the acrophases of both rhythms on each tested timespan were taken as estimates of circadian coordination.

Large intersubject differences characterized the median values of both activity, whose median was 39.8 movements per min [IQ, 19.3 to 54.7], and skin surface temperature, whose median was 35.2°C [34.7 to 35.8]. The rest-activity rhythm displayed a dominant 24-hour period for all 55 subjects. In contrast, the skin surface temperature displayed a dominant period in the circadian range for 39/55 subjects (71%), and in the circahemidian range for 13/55 subjects (27%). Three out of 55 subjects (5%) displayed atypical patterns, one for both variables, due to trans meridian travel; one with a dominant temperature period of 6 hours; and one with an unstable temperature pattern. We categorized the subjects as belonging to *Class A* (circadian rhythms in both variables), *Class B* (circadian activity and circahemidian temperature) or *Class C* (atypical rest-activity and/or temperature patterns, not shown; Figure 5). For *Class A*, the median circadian acrophases were located at 14:40 for activity (with individual acrophases ranging over 7 hours 44 min, from 12:41 to 20:25), and at 3:33 at night for skin surface temperature, yet with intersubject differences spread over 24 hours. Thus, the skin surface temperature acrophase occurred at night (22:01 to 7:00) for 31 subjects and during day-time (7:01 to 22:00) for 8 subjects in Class A (*P*<.001 from exact Fisher test). As a result, the median time interval between both rhythm acrophases was 11 hours 47 min, yet it had a wide range from 7 min to 12 hours among the 39 Class A subjects.

**Figure 6:**
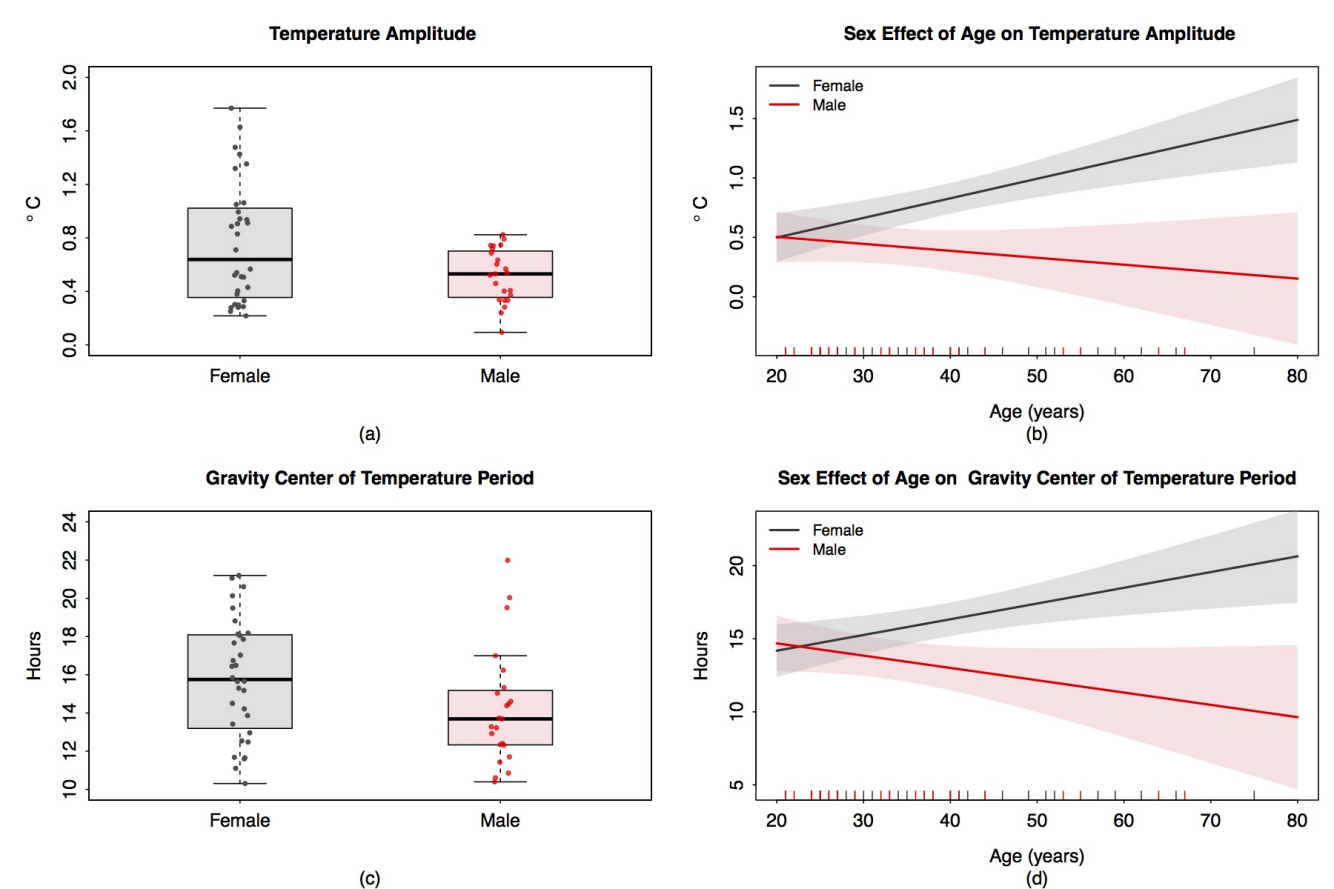
Sex and sex-age dependencies of circadian amplitude (upper row) and spectrum gravity center of temperature time series (lower row) in 55 healthy subjects. (a) Box plot of temperature amplitudes of the estimated main harmonic for females (left, N=32) and males (right, N=23); (b) Sex-specific effect of age on temperature amplitude shown by estimated regression line with 95% confidence bands. The vertical dashes along the horizontal axes show corresponding age of each subject. (c) Box plot of the estimated gravity center of temperature spectra for females (left, N=32) and males (right, N=23). (d) Sex-specific effect of age on the gravity center of temperature spectra shown by estimated regression line with 95% confidence bands.

**Figure 7:**
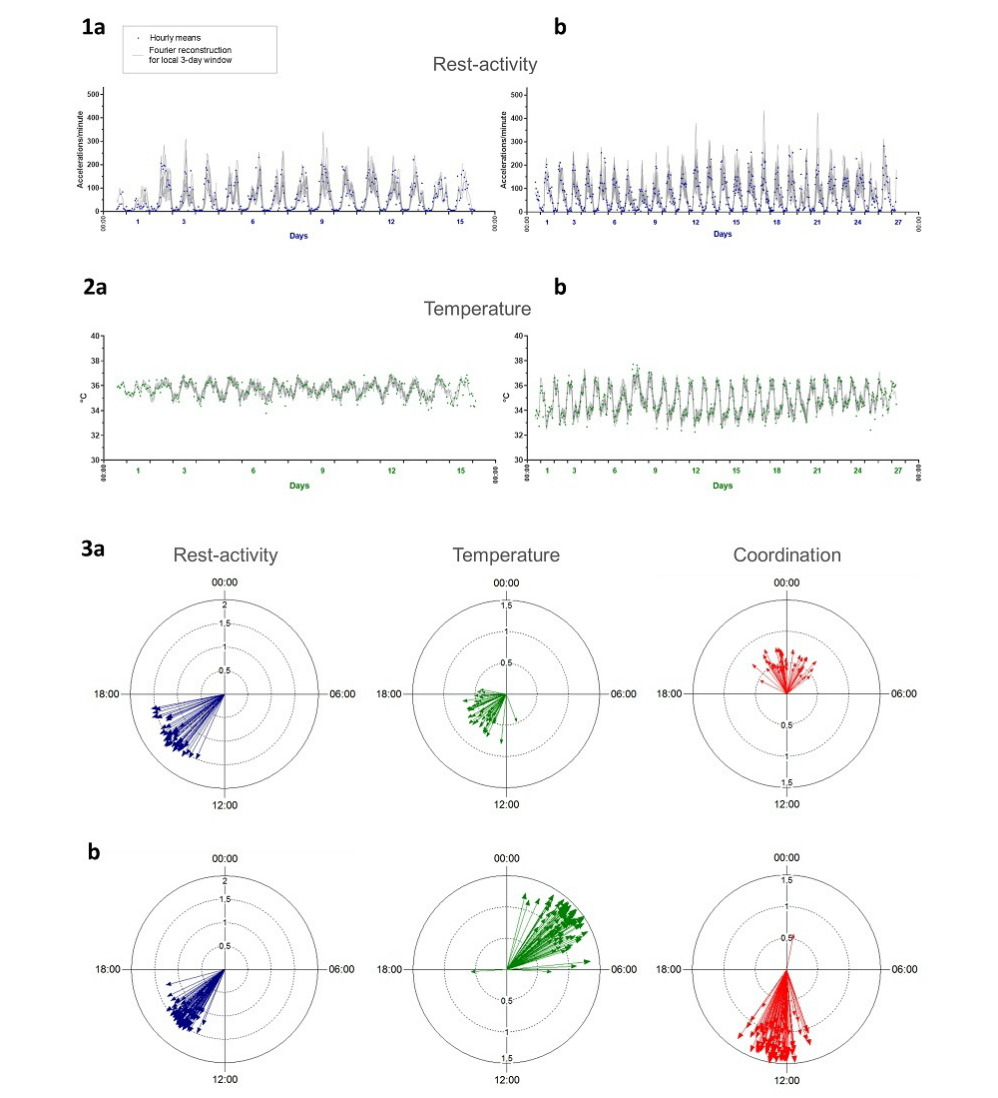
Inter- and intrasubject differences in circadian patterns in rest-activity and chest surface temperature as illustrated in 2 cancer patients (a and b), with chronograms (panels 1 and 2) and polar plot representations of amplitude-acrophase vectors (panel 3).

### Sex and Age Moderation of Temperature but Not Activity Rhythms

Statistically significant pairwise Spearman correlations with |r|≥0.4 were identified for median values and their corresponding dominant rhythm amplitudes for both activity (*r*=.77) and temperature (*r*=.49). Sex jointly with age had a selective influence on the rhythmic organization of temperature, but not that of rest-activity in the 55 healthy subjects. This was statistically validated by multivariate regression analysis of amplitude and spectral gravity center (or “mean period”) of temperature, as response variables, with age and sex as covariates (Figure 6). Females displayed larger temperature amplitude than males (two-sample *t* test, *P*=.005; Figure 6). Females also had larger values than males in the estimated gravity center of their temperature spectrum (two-sample *t* test, *P*=.03). Overall, the results indicated that females tended to mostly display dominant 24-hour rhythm periods with large amplitudes, whereas 12-hour rhythms tended to predominate in males (Figure 6). The interaction between sex and both temperature rhythm parameters was mostly apparent beyond 35 years. Regression analysis showed that sex, age, and the interaction term sex*age had a significant effect on temperature amplitude (with P values=.04, <.001 and .002, respectively) and on the gravity center of the temperature spectra (with P values of .03, .004, and .003, respectively; Figure 6).

### Circadian Coordination in Cancer Patients

A total of 7 male and 5 female patients tested the platform for a median of 19 days, ranging from 4 to 29 days. All the patients had previously received 1 or more chemotherapy protocols for metastatic gastrointestinal cancer, including colorectal (N=5), pancreatic (N=3), liver (N=2), stomach (N=1), or anal (N=1) cancer. Moreover, 5 patients had co-morbidities (see Multimedia Appendix 1). Six patients received chronomodulated infusions, including 2 courses at home, while being telemonitored. Circadian patterns in rest-activity and thoracic temperature were identified in all the patients’ records, as shown by dominant 24-hour periods. Figure 7 depicts examples of circadian patterns in 2 cancer patients, a 53-year-old male (patient 1) on conventional chemotherapy with oxaliplatin-5-fluorouracil-leucovorin delivered during hospitalization for liver metastases from stomach cancer, on days 1 and 2 of the recording, and a 78-year-old male (patient 2) with lung and lymph node metastases from rectal cancer, whose treatment involved intravenous panitumumab and chronomodulated chemotherapy with irinotecan, oxaliplatin, 5-fluorouracil, and leucovorin at home, using a 4-channel programmable-in-time pump, from day 6 to day 9 of the recording. Usual awaking and retiring times were 7:50 and 21:50, respectively, for patient 1 and 7:15 and 23:00, respectively, for patient 2. Despite all patients belonging to Class A, large interpatient variations were found regarding the circadian amplitudes in both rest-activity (range 29.3 to 68.6 accelerations per minute) and temperature (0.17°C to 1.33°C). Individual rest-activity acrophases varied within a 5-hour range, occurring between 12:09 and 16:52. In contrast, the circadian acrophases of temperature were located between 22:01 and 07:00 at night for 8 patients, and at daytime for another 4 patients. Intersubject variations were also revealed with dichotomy index I<O, whose individual median values ranged from 88.2% to 99.7%, with rather low estimated intrasubject coefficients of variations of 0.3% to 6.4%. Interestingly, the circadian acrophase of temperature was located at daytime for 3 of 6 patients with an I<O under 97.5%, but at nighttime for 5 of 6 patients, whose I<O was over 97.5% (*P*<.001 from exact Fisher test).

### Relations Between I<O and Chest Temperature Timing Among the Study Population

An I<O of 97.5% or less was found in 9 of the 51 subjects, with a 24-hour temperature rhythm (18% of Class A), and in 1 of the 13 subjects, with a 12-hour temperature pattern (8% of Class B). We did not consider further the 3 Class C subjects (1 with a high I<O, and 2 with a low one). Interestingly, the average circadian acrophase of chest temperature was localized at daytime, ie, between 7:01 and 22:00 for 6 of the 54 subjects with a high I<O value (11%), as compared with 6 of the 10 subjects whose I<O was low (60%); *P*<.001 from exact Fisher test). Indeed, having a high I<O enabled the accurate prediction of a chest temperature occurring at night, whereas no reliable prediction of temperature rhythm timing could be made in the subjects whose I<O was low (see Multimedia Appendix 4).

## Discussion

### Effective Mobile eHealth Platform

A mobile eHealth platform (PiCADo) consisting of a BLE chest sensor and a pocket-sized BT/BLE/GPRS gateway effectively measured body accelerations, 3D orientation, and upper chest temperature every 1-5 min for prolonged timespans, teletransmitting them to a central server every 24 hours. This interval hat can be reduced to 1 hour. In 67 healthy people or cancer patients, this technology revealed large interindividual differences in circadian coordination that was captured in real time during their daily routine. The multidimensional platform and its capabilities to combine electronic patient-reported outcomes (PROs) with circadian rhythm monitoring and body weight measures meet the expectations of the American Society for Clinical Oncology, regarding the future of eHealth technologies in cancer medicine [52]. Thus, the weekly transmission of electronic PROs significantly improved overall survival in 2 randomized trials conducted in cancer patients, indicating this information might elicit initially unplanned interventions and/or modify patient’s engagement with an oncologic benefit equivalent to that of the addition of an active drug [53,54]. The PiCADo platform provides a novel framework for the further integration of circadian rhythms and other parameters into proactive timely care interventions and the ready assessment of their efficacy. Such technology enables a novel systems approach for a coordinated medical and care logistics fit for the management of chronic disease and cancer patients, so called Domomedicine [26,55].

### Technology and Compliance

The reliability and acceptance of the PiCADo platform is illustrated from the records obtained in the subjects from 2 countries, including 55 healthy ones and 12 cancer patients at home or during their usual activities, for prespecified durations of 4 up to 30 days. Indeed, valid time series were extracted from the server, and amenable to longitudinal statistical analyses. These results are in line with those obtained previously in 31 cancer patients through a fixed internet platform within the inCASA European project [26]. The current PiCADo platform was developed to bypass the limitations of the inCASA platform, through mobile technology, and multirhythm monitoring, including skin temperature. Its specifications aimed at the broad integration of circadian rhythms into medicine, as a potential new information for triggering progress in the proactive management of cancer and chronic diseases.

Further real-life tests (not shown here) indicated the reliability of combining such rhythm telemonitoring with other physiologic or patient-reported outcomes parameters, such as body weight or self-rated symptoms, through additional BLE-connected devices.

### Circadian Coordination of Healthy People or Cancer Patients During Their Daily Routine

This study has highlighted the consistency of the 24-hour patterns in both rest-activity and thoracic skin surface temperature from one day to the next both in healthy and cancerous patients. However, marked intersubject differences were found regarding the dominant period, the spectrum central gravity, and the amplitude and acrophase location of temperature. More specifically, nearly 1/4 of the healthy subjects had a prominent 12-hour rather than 24-hour periodic temperature rhythm. Sex and age, but not weight, were influential on the temperature rhythms, as discussed below. Among the 39 healthy subjects with a predominantly circadian chest temperature rhythm, the acrophase was located at night, as expected [33,56,57], for 79% (31/39), or at daytime for 20% (8/39). Indeed, it is known that skin surface temperature usually increases at night, thus resulting in heat dissipation and the core body temperature drop that has been associated to sleep triggering [57]. In contrast, intersubject variability was limited for rest-activity, with all the 67 subjects displaying a dominant circadian rhythm, and 97% (65/67) of them having an acrophase of activity located in the afternoon or early evening. Consistently, only 5 of 55 healthy subjects had a dichotomy index I<O between 97.5% and 95.5%, a rate in good agreement with a prior study using wrist actigraphy in 182 young subjects [27]. Half of the 12 cancer patients had an I<O ranging between 97.5% and 88.7%, ie, the same rate as that previously reported using wrist actigraphy in 436 cancer patients [22,27]. This suggests that there is consistency of both recording methods regarding I<O estimation. There were statistically significant relations between temperature period and amplitude on the one hand, and sex, age, and I<O on the other hand; however, no such correlations were found for the circadian rest-activity parameters derived from the spectrum. Moreover, the circadian acrophase of thoracic temperature occurred at nighttime, thus indicating physiologic circadian coordination, for 48 of the 54 subjects (89%) whose I<O exceeded 97.5%, whereas it was located at daytime for 6 of the 10 subjects (60%) with a lower I<O. These findings suggest that low I<O values could reflect nonphysiologic circadian coordination. Hence, temperature monitoring significantly increased the information already provided by rest-activity regarding CTS function, as suspected in pilot studies [44,45].

### Sex and Age as Important Determinants of Circadian Coordination and Timing

Here, women aged >35 years tended to have robust 24-hour rhythms in their temperature, with larger amplitudes as compared with those in males. This apparent difference between circadian rhythms in temperature and activity has a neuroanatomic basis, which has been demonstrated in rodents [58]. Thus, the rest-activity and temperature rhythms were shown to be generated by the neurons located in the caudal [59] and subparaventricular zone of the suprachiasmatic nuclei [60,61], respectively. As body temperature is an important driver of both the resetting and the coordination of peripheral clocks [8], its rhythm could play a critical role for the circadian timing of medications [13]. The larger amplitude of the circadian rhythm in temperature shown here for women supports the hypothesis that circadian timing of medications is even more critical in women as compared with men. Moreover, larger circadian amplitudes have also been found in females, compared with males, for cortisol secretion, another key driver of peripheral clocks and clock-controlled metabolism and cell-cycle determinants of drug effects [5,62]. Two separate clinical trials uncovered that the optimal timing of a multidrug chronomodulated chemotherapy protocol could lag 6 hours behind in women as compared with men with the same cancer type [63,64]. Such differences in circadian amplitudes and time lag between males and females were similar in cancer patients and in mouse models [65,66]. The sex-related differences in CTS that were discovered here could explain why the same multidrug cancer chronotherapy protocol improved response rate, progression-free survival, and overall survival in men, but not in women with metastatic colorectal cancer, independently of all other prognostic factors within a meta-analysis of 3 randomized international clinical trials involving 842 patients [28].

### Study Limitations and Current Perspectives

The main limitations of our study beyond the technological possibilities of the eHealth platform was its exploratory nature, with unbalanced sex and age distribution, as well as a limited number of cancer patients. We did not assess chronotype via an established questionnaire [67], or working hours, although the temperature and activity records provided an objective and quantitative phase assessment [68,69]. Other indicators of circadian function, such as cortisol or melatonin rhythms or Dim Light Melatonin Onset [70], were not determined, nor did we investigate clock genes polymorphisms [71]. Indeed, genetic variants of human clock genes have been shown to be associated with phenotypic differences, which could allegedly impact disease processes, including cancer [72]. Moreover, the various biomarker rhythms of circadian function can be differentially altered by disease processes [73].

### Comparison With Prior Work

However, the technology supporting the platform here presented allows to fully integrate the new information brought about by circadian rhythms, jointly with repeated self-assessed symptoms and other measurable parameters. The PiCADo platform represents an answer to the limitations of the current hospital-centered care system, which was designed for responding to acute medical events, rather than for managing the long-term medical care required for cancer and other chronic diseases [74-76]. The latter illnesses not only represent the vast bulk of health care payload in Western countries, but also their management is suboptimal, given the current hospital logistics constraints [77,78]. This is especially true, as disease and treatment response dynamics vary from patient to patient, with most events occurring at home and remaining unnoticed, while impacting daily life, and eventually cumulating and leading to emergency hospitalizations. In the previous inCASA study, we showed the reliability of a fixed internet platform for the remote monitoring of self-rated symptoms, body weight, and rest-activity rhythm in cancer patients, and the rather good patient compliance. Moreover, such remote monitoring provided early warning signals of patient deterioration at home that resulted in emergency hospitalization 3 days later. Circadian disruption played a prominent role in the determination of such early warning signaling [26]. Here, we aimed at improving circadian timing disorders detection through combining rest-activity and temperature monitoring, while being also able to integrate self-rated symptoms and body weight measurements, using a mobile, rather than fixed platform. We now believe that such PiCADo eHealth platform could shift the current hospital-centered system of care toward a patient-centered system promoting biomedical progress. This would involve the safe delivery of care and support treatments at home or during the patient’s daily activities, through adjusting cancer therapies to circadian rhythms, ie, chronotherapy [26,79-81]. PiCAdo further provides an ongoing monitoring system of the patient’s well-being through developing forecasting analytical methodology integrating multiple sources (patient-reported outcomes, circadian rhythms in activity and temperature, physiological measures).

### Conclusions

We have shown that such a mobile eHealth circadian platform allows automatic and noninvasive monitoring of precise circadian parameters in nonhospitalized healthy subjects and cancer patients. Preliminary analyses point to large and unsuspected intersubject variabilities, which may be of great importance when administering treatments and preventing emergency situations. Hence, it deserves further testing as a tool for the real-time assessment of the CTS of humans of both sexes in various conditions, where 2 types of studies are currently in progress or about to start, respectively, in France and in the United Kingdom. The platform is being used for the determination of the impact of the occupational schedule on circadian function in the CIRCADIEM study, whereas the service rendered by this circadian eHealth platform is to be investigated further in cancer patients in the IDEAs study. Clearly, such system now needs assessment over months and in large patient cohorts within a prospective clinical trial. eHealth circadian monitoring may indeed be of preventive and curative interest in a number of situations involving chronic conditions [82].

## Appendix

Multimedia Appendix 1 Patient characteristics.

Multimedia Appendix 2 Intersubject variabilities in main rhythm parameters of healthy subjects in Cohort 1.

Multimedia Appendix 3 Intersubject variabilities in main rhythm parameters of healthy subjects in Cohort 2.

Multimedia Appendix 4 Polar plots of mean temperature acrophases in study population according to dichotomy index I<O value.

## Abbreviations

BLE: Bluetooth Low Energy
BT: Bluetooth
CTS: circadian timing system
eHealth: electronic health
GPRS: general packet radio service
IQ: interquartiles
IQR: interquartile range
PRO: patient-reported outcome
