# Supplementary material for: Relevance of a Mobile Internet Platform for Capturing Inter- and Intrasubject Variabilities in Circadian Coordination During Daily Routine: Pilot Study
Source: J Med Internet Res. 2018 Jun 11;20(6):e204. doi: 10.2196/jmir.9779 (PMC6018238; doi:10.2196/jmir.9779)
Supplement: Multimedia Appendix 1 [file jmir_v20i6e204_app1.pdf]

Multimedia Appendix 1: Patients characteristics

**Characteristics**	
**Age (years)**	
Median (range)	61 (47-83)
	**Number of patients**
**Sex**	
Male / Female	7 / 5
**WHO Performance Status**	
0 / 1	6 / 6
**Primary Tumor Site**	
Colo-rectal	5
Pancreas	3
Liver	2
Other GI	2
**Number of metastatic sites**	
0	1
1	4
≥ 2	7
**Co-morbidities**	
None	7
1	2*
≥ 2	3**
**Prior cancer surgery**	
None	8
Primary tumor only	1
Primary tumor and metastases	3
**Prior chemotherapy**	
Adjuvant and metastatic	2
Metastatic only	10
**Number of prior chemotherapy protocols for metastatic disease**	
One	4
≥ 2	8

* Hypothyroidy (N=1); glaucoma (N=1)

** osteoarthrosis and venous thrombosis (N=1); Lynch syndrome and gastroesophageal reflux (N=1); multiple cardiovascular and metabolic diseases (N=1: lower limbs arteriopathy, atrial fibrillation, valvulopathy, ischemic heart disease, arterial hypertension; type 2 diabetes, dyslipidemia)
