# Supplementary material for: Relevance of a Mobile Internet Platform for Capturing Inter- and Intrasubject Variabilities in Circadian Coordination During Daily Routine: Pilot Study
Source: J Med Internet Res. 2018 Jun 11;20(6):e204. doi: 10.2196/jmir.9779 (PMC6018238; doi:10.2196/jmir.9779)
Supplement: Multimedia Appendix 2 [file jmir_v20i6e204_app2.pdf]

Multimedia Appendix 2: Intersubject variabilities in main rhythm parameters of healthy subjects in Cohort 1.

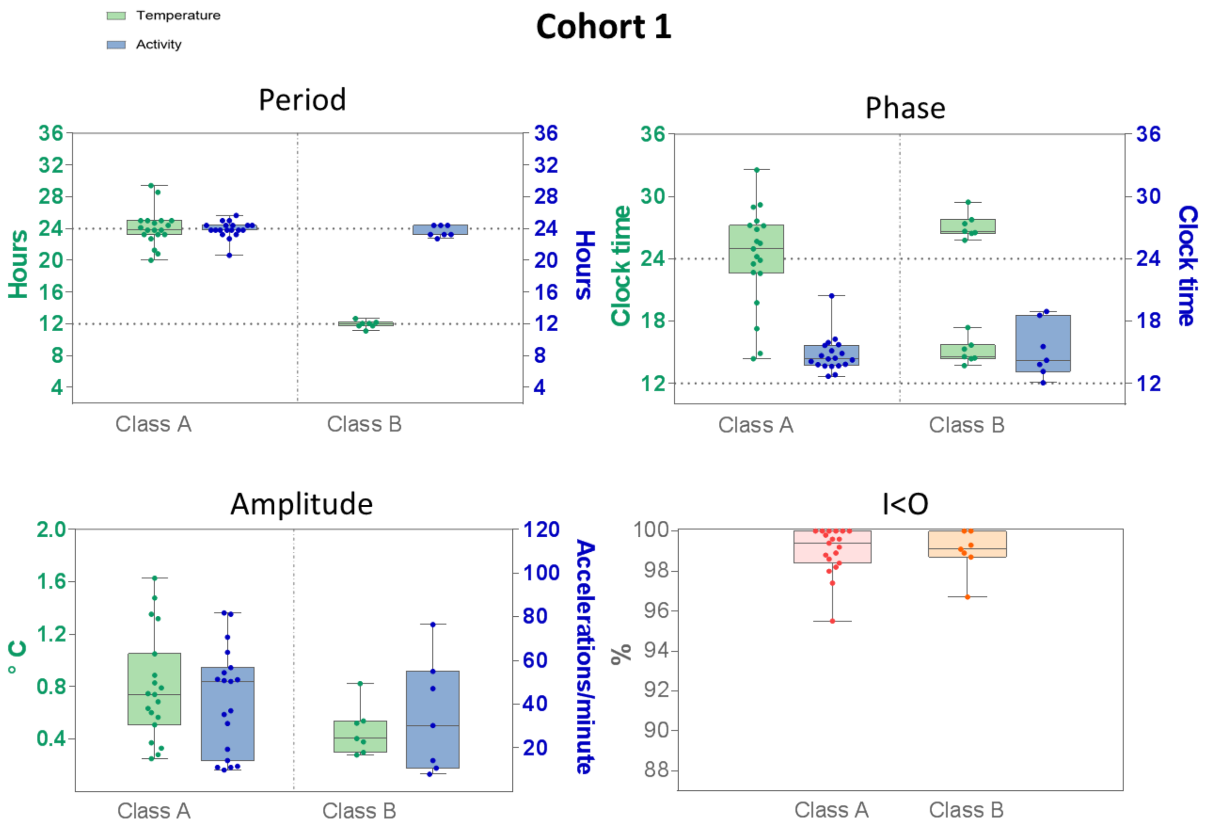

Median, interquartiles, range and individual values of dominant periods and corresponding amplitudes and acrophases of temperature (green) and rest-activity (blue), based on spectral analysis of time series over the whole timespan. Healthy subjects are classified according to dominant temperature period of ~24 h (Class A) or ~12 h (Class B), while all of them display a dominant 24-h rhythm in activity. The bottom right panel depicts the distribution of the dichotomy index I<O of the rest-activity pattern in Class A or B healthy subjects.

Comment: Median and range of both skin surface temperature and activity varied largely according to individual subject. According to spectral analysis of the whole time series, a dominant circadian rhythm was identified for 19 subjects for skin surface temperature (67.8%), with estimated circadian periods, ranging from 20 to 29.4 h. Seven subjects had a dominant 12-h rhythm (25%) and 2 subjects

had no identifiable rhythmic pattern (7.1%). Thus, the skin surface temperature rhythm was synchronized to the 24-h environment for 26 subjects (92.8%), yet with amplitudes ranging from 0.25 to 1.63 degrees C (>6-fold intersubject differences) and acrophases spread from 00:12 at night to 14:20 (differing by up to 9 h and 52 min). In contrast a dominant circadian period was identified for rest-activity in the 28 subjects, with amplitudes ranging from 7.3 to 81.7 accelerations per min (11-fold differences) and acrophases varying from 12:05 to 20:42 (i.e. by up to 8h37 min). Individual I<O values ranged from 95.4 to 100%, with 21 subjects (75%) displaying values > 98.6.
