# Supplementary material for: Relevance of a Mobile Internet Platform for Capturing Inter- and Intrasubject Variabilities in Circadian Coordination During Daily Routine: Pilot Study
Source: J Med Internet Res. 2018 Jun 11;20(6):e204. doi: 10.2196/jmir.9779 (PMC6018238; doi:10.2196/jmir.9779)
Supplement: Multimedia Appendix 3 [file jmir_v20i6e204_app3.pdf]

Multimedia Appendix 3: Intersubject variabilities in main rhythm parameters of healthy subjects in Cohort 2.

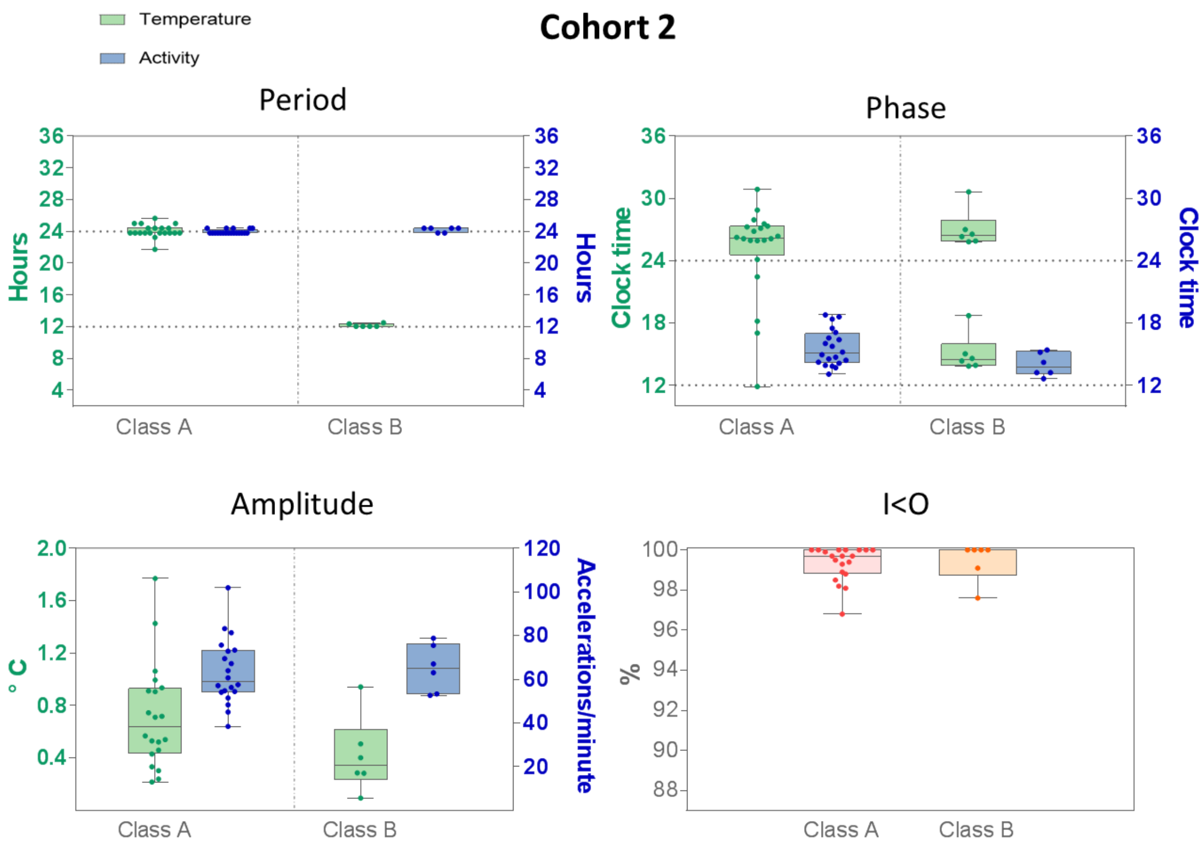

Median, interquartile range and individual values of dominant periods and corresponding amplitudes and acrophases of temperature (green) and rest-activity (blue), based on spectral analysis of time series over the whole timespan. Healthy subjects are classified according to dominant temperature period of ~24 h (Class A) or ~12 h (Class B), while all of them display a dominant 24-h rhythm in activity. The bottom right panel depicts the distribution of the dichotomy index I<O of the rest-activity pattern in Class A or B healthy subjects.

Comment: Results from spectral analysis on 7-day or 21-day time series, from Cohorts 2a and 2b respectively, were consistent with those obtained for the 4-day time series in Cohort 1 (**see Multimedia Appendix 1**). Thus, 20 subjects (74.1%) displayed a dominant circadian period for skin surface temperature, ranging from 21.7 to 25.6 h, while 6 subjects had a dominant 12-h period

(22.2%), and one subject had no consistent temperature rhythm (3.7%). In contrast a circadian pattern in rest-activity was validated for the 27 subjects using both spectral analysis and dichotomy index determinations.
