# Supplementary material for: Relevance of a Mobile Internet Platform for Capturing Inter- and Intrasubject Variabilities in Circadian Coordination During Daily Routine: Pilot Study
Source: J Med Internet Res. 2018 Jun 11;20(6):e204. doi: 10.2196/jmir.9779 (PMC6018238; doi:10.2196/jmir.9779)
Supplement: Multimedia Appendix 4 [file jmir_v20i6e204_app4.pdf]

Multimedia Appendix 4: Polar plots of mean temperature acrophases in study population according to dichotomy index I<O value.

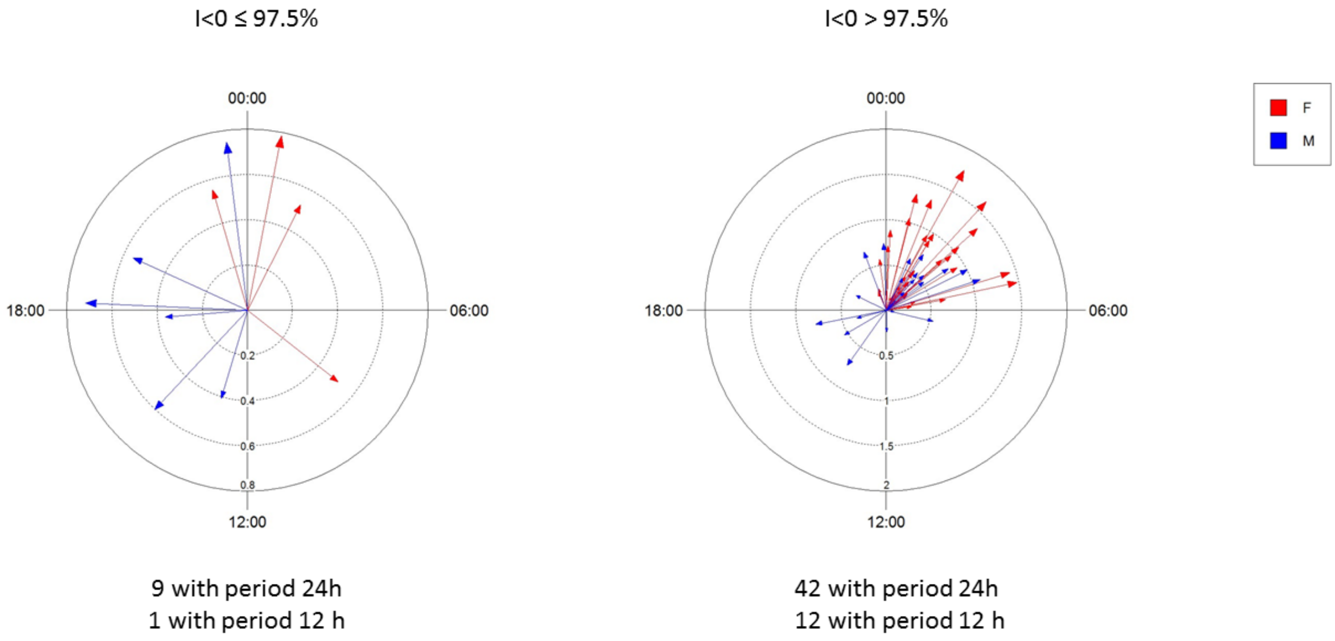

Temperature amplitudes (vector length) and acrophases (arrows pointing toward timing) in 10 subjects with I<O of 97.5 % or less (*left*) and in 54 subjects with an I<O above 97.5% (*right*).

The 3 subjects in Class C are not considered, since their temperature acrophase was unstable over the recording timespan (see text).
